# The occurrence of fungi from burn wound patients and antifungal susceptibility patterns: a cross-sectional study in Lusaka, Zambia

**DOI:** 10.4314/ahs.v23i3.58

**Published:** 2023-09

**Authors:** Kapembwa Sikwewa, Paul Simusika, Mulowa Mumbula, Darlington M Mwenya, Chungu Mandona, Gina Mulundu

**Affiliations:** 1 University of Zambia, School of Medicine, P.O. Box 50110, Lusaka, Zambia; 2 University of Zambia, School of Medicine, Department of Pathology and Microbiology, P/Bag RWX1, Lusaka, Zambia; 3 University Teaching Hospital, Department of Pathology and Microbiology, P/Bag RWX1, Lusaka, Zambia

**Keywords:** Burns, *Candida albicans*, CLSI, fungal infection, Susceptibility patterns

## Abstract

**Background:**

Fungal opportunistic infections in burn wound patients are among the leading cause of morbidity and mortality. Attention remains focused on preventing bacterial infection at the expense of increasing fungal infection in burn wound patients.

**Objective:**

To determine the occurrence of common fungi in admitted burn wound patients and their environment: and their antifungal susceptibility patterns at the University Teaching Hospitals, Lusaka, Zambia.

**Methods:**

This laboratory-based cross-sectional study enrolled a total 101 participants whose pus swab specimens were collected from their burn wounds as well as 50 environmental swabs collected from strategic points. Wet mount, gram stain, culture on Sabouraud dextrose agar, Corn meal agar and Germ tube were used to identify possible fungal isolates. Agar based disc susceptibility test was carried out using fluconazole. Data was analysed using Excel and STAT version 14.

**Results:**

Median age was 3 years and median burn % of TBSA was 18 in participants' who had burn wound fungal infection and consisted of 3 males and 6 females. Organisms isolated included *Candida albicans* from 8(7.9%) participants and 2(4%) from 50 environmental swabs. 1(1%) *Candida spp* was isolated from pus swabs. Out of the total 11 *Candida* isolates, 4 (36.4%) were susceptible to fluconazole and 7 (63.6%) were resistant.

**Conclusion:**

The isolation of *Candida albicans* and *Candida spp* from burn wound patients and the hospital ward environment suggests presence of fungi in burn wound patients and hospital ward environments. *Candida* isolated showed varying susceptibility patterns to fluconazole.

## Introduction

Burn patients are continually at risk of contracting fungal infections as they may be exposed based on the degree of burned total body surface area (TBSA). Burn wounds may result from damage to the skin caused by non-mechanical sources such as heat, electricity, chemicals, and nuclear radiations 1. Burn Wound Infection (BWI) remains a major public health problem and a cause of devastating trauma that can induce immediate immunosuppression which may attract predisposition to infectious complications. There has been noticeable improvement in outcomes of burn wound patients, attributed to fluid resuscitation, nutritional support and general advanced use of barrier nursing and antimicrobial agents 2. This however, is still in contrast to opportunistic fungal infections in burn wounds patients that have been reported to be on an upswing 2,3,4,32.

The skin provides an important immune barrier and if damaged, it may result in suppression of local and systemic host cellular and humoral immune responses that can destroy circulatory immune factors and result in severe opportunistic infectious complications 5,6,7. Burn wound patients with increased percentage of total body surface area burned (%TBSA) and nursed in intensive care unit for a long-time are at higher risk of fungal infections 6,8,9. Although it is known that some fungi might colonize human beings as part of normal flora, fungi still opportunistically infect burn wounds, resulting into Fungal Wound Infection (FWI) 10]which this study was concerned with. Reports from burn centres around the world show variations in the incidence of fungal infections 8,11,12,13. *Candida albicans* which frequently colonizes burn wounds is associated with the surrounding environment or as commensals and often causes infections within the second week of thermal injury, after patients may have received multiple antimicrobials 12,14,15. In resource poor settings, fungal infections related to burn wounds often go undiagnosed due to several factors that include limited clinical awareness and inadequate mycological laboratory facilities to distinguish them from bacterial opportunistic infections. The study aimed at investigating the occurrence of common fungi associated with admitted burn wound patients and their environment and determined antifungal susceptibility pattern of such fungi at the University Teaching Hospitals, Lusaka, Zambia.

## Materials and methods

### Study site

The study was conducted on swab samples from 101 participants with an extra 50 environmental swabs from a dedicated burn unit of the Adult Hospital of the University Teaching Hospitals, Lusaka, Zambia. This hospital serves as the main referral hospital in Zambia.

### Specimen collection

Patients who presented with burn wounds were recruited into the study based on the inclusion and exclusion criteria. Consent was obtained from participants or their guardians prior to obtaining specimens. The specimens were carefully obtained from the burn wounds by rolling the tip of the swab on its side for one full rotation on the surface and edges of burn wound without touching intact skin to avoid possible contamination. The first specimen was collected on day eight after admission, with a follow up specimen being collected on day fourteen. In addition, environmental swabs were collected from surfaces of bath tubs, mattresses, linen and the floor.

### Culture of pus and environmental swabs

Swabs collected were cultured in duplicate on Sabouraud dextrose agar plates (Mast diagnostic Ltd, Merseyside, United Kingdom). One culture plate was incubated aerobically at 37°C for 1 week and checked for any growth daily. The other culture plate was incubated at 25°C (room temperature) for 2 weeks and checked daily.

### Identification of isolates

All culture growth suggestive of fungi was subjected to gram stain.

### Germ tube test for yeast

Wet mounts in light microscopy were used to look for any sprouting yeast cells and any cells exhibiting tube-like outgrowths.

### Corn meal agar

Corn Meal Agar Medium was used to check for chlamydospore production by *Candida albicans*. These would appear as thick-walled mycelia bearing a ball like cluster of budding cells. Yeast colonies grown on Saboraud dextrose agar (Mast diagnostic Ltd, Merseyside, United Kingdom) were sub cultured using sterile straight wire and streaked (horizontal furrow) onto corn meal agar (Oxoid Ltd, Basingstoke, Hampshire, United Kingdom). A flamed-sterilised cover slip was placed over the line of inoculum. The streaks were examined microscopically for any growth using a low power objective after 48 hours of incubation at 22^0^C.

In order to differentiate *C. albicans* from *C. dubliniensis*, pure candida species isolates were inoculated on sabouraud dextrose agar and incubated at 42°C for 48 hours. On plates where growth was obtained at 42°C, the isolate was identified as *C. albicans* and not *C. dubliniensis* because *C. albicans* is able to grow at higher temperatures such as 42°C 34,35

### Antifungal susceptibility testing

The antifungal susceptibility testing in this study was performed on *Candida albicans* isolates using the agar-based Disc diffusion method by plating the inoculum on Muller Hinton (Oxoid Ltd, Basingstoke, Hants, United Kingdom) supplemented with 2% glucose (Skylabs, Springfield, South Africa) and 0.5 microgram/millilitre methylene blue (Himedia, Mumbai, India) in 130 mm diameter plates according to clinical laboratory standard institute guidelines.

The plates were inoculated by dipping a sterile swab into an isolate suspension adjusted to 0.5 McFarland standard units (10^6 cells/ml). The standardised inoculum was then streaked across the surface of the agar plates and allowed to dry at ambient temperature for 15 minutes. Antifungal discs were then placed on the inoculated plates. The susceptibility test results were determined after 24 and 48hours of incubation at 35^0^C. The following antifungal agents were used: 10µg fluconazole, 20µg Amphotericin B and 30µg Ketoconazole (Himedia, Mumbai, India). These antifungal agents are commonly used for the treatment of fungal infections in Zambia. Susceptibility of isolates to Amphotericin B and Ketoconazole could not be interpreted due to luck of CLSI guidelines. However, all specimens were preserved in vials containing 50% glycerol solution and frozen at −80°C for future reference. Zone diameter breakpoints for disc diffusion method for fluconazole: Sensitive ≥17, Intermediate 14 – 16, Resistance ≤13.

### Data processing and analysis

Data generated from the study was analysed using Excel and STATA version 14. All continuous variables were tested for normality using the Shapiro – Wilk test. Median and interquartile range were used as descriptive statistics for data which was not normally distributed. Mann-Whitney test was used to analyse asymmetrical data and multiple logistic regressions was used to determine factors such as age, burn percent of TBSA and HIV status associated with burn wound fungal infection.

## Results

In this study, burn wounds swabs were collected from a total of 101 participants who met the inclusion criteria and consented to the study. Of the total 101 burn wounds, 8 were infected with *Candida albicans* and 1 was infected with *Candida spp*. An additional 50 environmental swabs were also collected from various surfaces in the wards. Of these, 2 had *Candida albicans*, isolated from a floor swab in the bathroom and the other from the floor in the burn's unit in ward G 12.

Table 1 below shows that median age was 3 years and interquartile range was 1.3 – 21 in participants with fungal wound infection. The median burn percentage of TBSA in participants where fungi was isolated was 18 % with interquartile range 15 – 26 while median burn percentage of TBSA in participants with no fungal isolates was 12.5 % with interquartile ranges of 8 – 20.5. Of the total 9 participants with burn wound fungal infection, 1(11%) was HIV positive while 8 (89%) participants were HIV negative, 6 (67%) participants were female while 3 (33%) were male.

**Table 1:** Fungal isolates per participant

Predictors	Fungi Isolated	Fungi not Isolated	P - Value
**Age** (years) M (IQR)	3 (1.3 - 21)	3 (1 – 5)	0.4485^MW^
**Burn % of TBSA**	18 (15 – 26)	12.5 (8 – 20.5)	0.1294^MW^
**HIV Status: Number (%)**			
HIV Positive	1 (11%)	1 (1%)	0.171^F^
HIV Negative	8 (89%)	91 (99%)	
**Gender: Number (%)**			
Female	6 (67%)	46 (50%)	0.489^F^
Male	3 (33%)	46 (50%)	

*Key: Fisher's exact test=F, Manny-Whitney=MW, Median=M, Interquartile, Range=IQR*

A total of 11 fungal organisms were isolated, 9 from burn wounds and 2 were isolated from the environment, 5 were found in G02 and another 5 was from G12. Only 1 was isolated in G21 as shown in table 2.

**Table 2:** Distribution of Fungal isolates

Surgical Ward	Number of fungal organisms		Name of fungal organism	
	Pus swabs	Environment	*C. albicans*	*Candida spp*
**G02**	5	0	4	1
**G11**	0	0	0	0
**G12**	3	2	5	0
**G21**	1	0	1	0
**G22**	0	0	0	0
**Total**	9	2	10	1

Most participants whose burn wounds were positive for *Candida* infection had burn percentage of total body surface area (TBSA) ranging from 16 – 20 (n=3 and 26+ (n=3) followed by 11 -15 (n=2) and 2 – 5 (n=1), confirming data that reported incidence of burn wound fungal infection to be more in individuals with greater TBSA, as shown in table 3.

**Table 3:** Burn % of TBSA of participants with *Candida* isolates

Burn % of TBSA	Frequency
1 – 5	1
6 - 10	0
11 - 15	2
16 - 20	3
21 - 25	0
26+	3
**Total**	9

**Figure 1:**
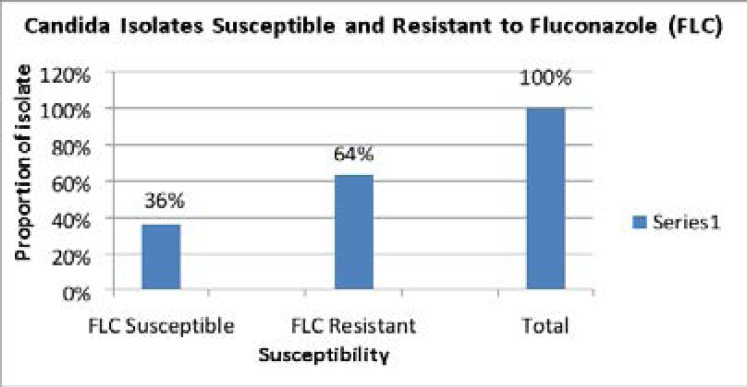
Susceptibility patterns of 11 Candida isolates, 4(36%) were susceptible to Fluconazole (FLC) and 7(64%) were resistant

Figure 1: Showing susceptibility results to Fluconazole (FLC)

The investigator-led backward stepwise regression approach was used to arrive at best predictor model. Table 4 below shows that Burn percentage of TBSA and HIV status were the best predictors of fungal infection of burn wound, with burn percentage of TBSA having a p-value = 0.097 and HIV status a p-value = 0.092 statistically significant at alpha 0.1 with confidence interval of 90%.

**Table 4:** Adjusted best predictor model for burn wounds and HIV status

Predictor	Odds ratio	P - Value	90% Conf. Interval
**Burn % of TBSA**	1.067901	0.097	1.000542 – 1.139794
**HIV Status**	11.85972	0.092	1.058589 – 132.8683

## Discussion

Fungi remain an important emerging cause of complications in burn wound infection (BWI), in both monomicrobial and polymicrobial infection 10,12,16.

This study involved 101 participants recruited, 53 (52.5%) being females [n=44 children, n= 9 adults] and 48 (47.5%) were males [n=45 children, n=3 adults]. The participant's age distribution ranged from 9 months to 63 years. The modal age group was 0 – 5 years old, indicating more children in the study. The modal range of burn percentage of TBSA were 16 -20 and 26+ in this study. One participant had burn percentage range of 1 – 5 representing a percentage of 11.1%.

Most fungal isolates came from specimens collected on day 14 after admission. Of the 9(8.9%) total burn wound isolates only 1 (1%) isolate was from the collection on day 8. These findings agree with earlier studies of burn wound infections such as an Indian study that documented 9 (75%) from 12 patients producing positive fungal growth during the second and third-week post burn 4. As in our study *Candida albicans* were found to be the most common pathogen in 8(88.9%) and *Candida spp*. being positive in 1(11.1%). In a similar study in Egypt at Cairo University burn centre by Ibrahim et al., fungal organisms were also isolated late in the second week post burn 17. Even with existing variations in findings of burn centres, the incidence of fungal opportunistic infection in burn individuals is still high at 6.3% to 15% 8.

In the current study, *Candida albicans* 8(7.9%) and 1(1%) *Candida spp*, were found to be the prominent fungi isolated. Fungi are widely distributed and are often implicated in open wound infections, as another study in America 8 documented. In that study of 6918 patients, 435 (6.3%) had positive fungal cultures isolated which included *Candida species* (371 patients; 85%), *Candida spp* (93 patients, 21%), Aspergillus (60 patients, 14%), and other mould (39 patients, 9.0%). A study determining the profiles of bacteria and fungi of burn wound infections in a tertiary care centre in India 18 found that of a total 218 isolates, 10 (4.59%) were fungal organisms and all were yeast. Among these, *Candida albicans* 5 (2.3%) was the most common isolate followed by a few combined *Candida species*.

General surveillance studies have equally reported presence of fungi in hospital environments 19. Reported fungi as being responsible for causing 9.5 % of hospital acquired infections, with *Candida species* alone causing 9% of the infections. Another hospital ward environmental study 13 reported 6(12%) of 50 patients admitted with thermal burns in the surgical unit had *Candida albicans* and 4(8%) non-albicans candida spp. Another Indian study from the National Mycology Referral Laboratory at a Postgraduate Institute of Medical Sciences, found that 25 severely burnt patients and their environment revealed fungal contamination by *Candida spp*, Penicillium, *Aspergillus* and *Fusarium* 20,21.

In our study, similar revelations of *Candida albicans* isolation from the environmental swabs collected from various surfaces in the burns unit especially in moist areas confirm the risk these organisms pose to not only burn wound patients but to all other patients admitted in hospitals. Children with burn wounds seem to be most at risk of fungal infections as shown by our results of the age group of 0-5 (n=6, 66.7%) with burn percentage of TBSA in the range of 16 – 20 (n=3, 33.3%) and 26+ (n=3, 33.3%). Having reported that burn wounds suppress immunity, it becomes more worrying for children with burn wounds whose immunity is not even fully developed, making this group of patients to be at heightened risk of fungal infection.

HIV infection (2%) among participants is another added risk to fungal infections in burn wound patients. HIV infections greatly lower immunity and thus makes burn wound patients more likely to be infected, not only with fungi but with other common environmental microorganisms too. Our study isolated fungi from 1(1%) of the participants who was HIV infected. Our statistical results confirm the risk association of HIV infection and burn percent of TBSA with risk of fungal infection (Burn % of TBSA: p-value=0.097, 90% CI 1.000542- 1.139794, HIV status: p-value=0.092, 90% CI 1.058589-132.8683) 1,5,4,22,33.

Successful clinical therapy of burn wound fungal infection has often been a challenge due to lack of mycology laboratories to do susceptibility testing and because of that some burn centres choose to use topical applications of antifungal agents to treat local colonization and fungal infections. Such treatments work but may also conceal deeper seated infections 23,24. Systemic use of antifungal agents therefore remains the main therapy of choice and administered dependant on the condition of the burn patient and possible laboratory confirmation of fungemia 1,8.

This study also considered subjecting the isolated fungal organisms to antifungal susceptibility testing to check susceptibility patterns of the commonly used antifungals in our environment. Susceptibility testing promotes accurate administration of antifungal agents and helps in monitoring emergence of antifungal resistance 25,26. Of the 11 *Candida* isolates tested against fluconazole, 4 (33.4%) were susceptible and 7 (63.6%) were resistant, a clear indication that there is resistance to fluconazole among the common fungi infecting burn wound patients. Two of the candida albicans isolates susceptible to fluconazole came from the environment swabs and the other two isolates were from burn wounds. This result echoes earlier findings in a study in Zambia by Sarenje et al., which observed multi-drug resistance in 5 *Candida* isolates, with *Candida albicans*, being the most resistant species to fluconazole and amphotericin B 27. *C. albicans* isolates were also found to be resistant to fluconazole in an Iranian study with patients responding poorly to treatment with these drugs 28,29,30,31.

## Limitations

The study might have suffered limitations arising from loss of burn wound patients who were admitted for less than a week and those who left against medical advice. Failure to collect and examine burn wound tissue for histology deprived the study useful data. Lack of guidelines on interpretation of zone of inhibition for antifungal disc diffusion methods for ketoconazole and amphotericin B also made it difficult to interpret the susceptibility patterns of the two antifungal medicines. Molecular methods were not used due to limited financial resources.

## Conclusion

This study contributes to documented fungal infections being likely to occur in burn wound patients. *Candida albicans* and *Candida* spp are present in the hospital ward environments and pose risk of opportunistic infection to burn wound patients at UTHs. The isolation of other *Candida species* with varying susceptibility patterns to fluconazole pose higher resistance to other antifungal substances and may be associated with poor prognosis. Therefore, screening burn wound patients for fungi should be routine; and preventive measures should include frequent fungal surveillance and appropriate antifungal therapy.

## Figures and Tables

**Figure 2:**
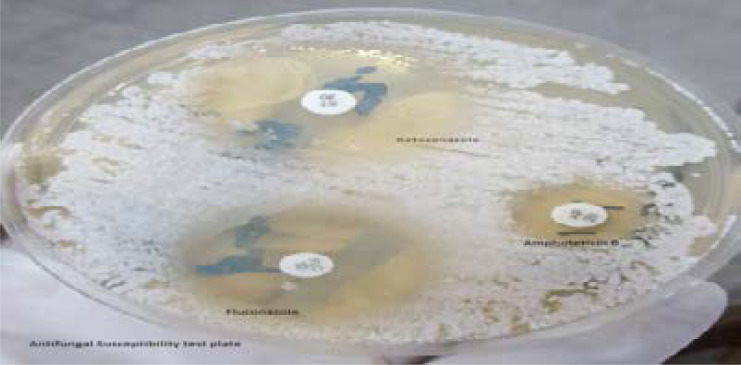
Antifungal Susceptibility test for *C.albicans* isolated from burn wound of a 1year 3months old girl on Mueller Hinton Agar + 2% Glucose + 0.5mcg/ml Methylene Blue Dye Medium (GMB

## Data Availability

Data generated and /or analysed in this study is available from the corresponding author on reasonable request.
